# Understanding disparities in cancer prognosis: An extension of mediation analysis to the relative survival framework

**DOI:** 10.1002/bimj.201900355

**Published:** 2020-12-14

**Authors:** Elisavet Syriopoulou, Mark J. Rutherford, Paul C. Lambert

**Affiliations:** ^1^ Biostatistics Research Group Department of Health Sciences University of Leicester Leicester UK; ^2^ Department of Medical Epidemiology and Biostatistics Karolinska Institutet Stockholm Sweden

**Keywords:** cancer inequalities, mediation analysis, natural indirect effect, regression standardization, relative survival

## Abstract

Mediation analysis can be applied to investigate the effect of a third variable on the pathway between an exposure and the outcome. Such applications include investigating the determinants that drive differences in cancer survival across subgroups. However, cancer disparities may be the result of complex mechanisms that involve both cancer‐related and other‐cause mortality differences making it difficult to identify the causing factors. Relative survival, a commonly used measure in cancer epidemiology, can be used to focus on cancer‐related differences. We extended mediation analysis to the relative survival framework for exploring cancer inequalities. The marginal effects were obtained using regression standardization, after fitting a relative survival model. Contrasts of interests included both marginal relative survival and marginal all‐cause survival differences between exposure groups. Such contrasts include the indirect effect due to a mediator that is identifiable under certain assumptions. A separate model was fitted for the mediator and uncertainty was estimated using parametric bootstrapping. The avoidable deaths under interventions can also be estimated to quantify the impact of eliminating differences. The methods are illustrated using data for individuals diagnosed with colon cancer. Mediation analysis within relative survival allows focus on factors that account for cancer‐related differences instead of all‐cause differences and helps improve our understanding on cancer inequalities.

## 1 INTRODUCTION

Survival after a cancer diagnosis varies considerably across subgroups. For instance, many studies have reported large disparities between socioeconomic groups that exist irrespective of the various approaches of defining socioeconomic groups (Danø, Andersen, Ewertz, Petersen, & Lynge, 2003; Ito et al., 2014; Jeffreys et al., 2009; Rachet et al., 2010; Rutherford, Andersson, Møller, & Lambert, 2015; Syriopoulou et al., 2017). Understanding the factors that drive survival differences can be challenging due to complex mechanisms that contribute towards disparities and the methodological challenges they induce.

Mediation analysis provides a useful tool for such settings as it can be applied to explore the role of a third variable (a mediator) that may be on the pathway between an exposure and the outcome (De Stavola, Daniel, Ploubidis, & Micali, 2014; Imai, Keele, & Tingley, 2010; VanderWeele, 2011). Thus, we can investigate whether differences in the mediator distribution are partly responsible for the variation between exposure groups. Mediation analysis helps to explore potential causal mechanisms of an observed association through an effect decomposition and under certain assumptions it allows the identification of the direct effect between an exposure and an outcome and the indirect effect due to the mediator (Pearl, 2001).

Another challenge with population‐based cancer data is the presence of competing events. The event of interest, which is usually death due to cancer, will never be observed for some patients due to an earlier death due to other causes. Cause of death information is often unreliable preventing a cause‐specific approach. The relative survival approach can be applied instead as it utilizes the expected mortality rates of a comparable group in the general population to represent mortality due to other causes for the cancer population (Dickman & Coviello, 2015; Ederer , Axtell, & Cutler, 1961; Pohar Perme, Stare, & Estève, 2012). Under assumptions, relative survival is interpreted as net survival, that is the probability of survival in a hypothetical world where the cancer of interest is the only possible cause of death (Pavlic & Pohar Perme, 2019).

Relative survival is a useful measure for disentangling the exposure–outcome association as it enables us to focus on cancer‐related differences instead of all‐cause differences. All‐cause survival differences are the result of complicated mechanisms that involve both cancer‐related and other cause factors. Thus, it may be easier to investigate the underlying determinants of cancer‐related differences and identify interventions of eliminating them. Such interventions would reduce the cancer mortality rates of those with worse prognosis but would have no impact on other cause mortality rates.

We extend mediation analysis to the relative survival framework and utilize regression standardization to obtain the marginal survival and related functions. We assess how much of the differences between exposure groups can be explained by differences in the mediator distribution, and we estimate the impact of removing differences in relative survival and the mediator distribution. For the interpretation of these measures as causal, standard mediation analysis assumptions are now extended in the relative survival framework and they need to hold both for cancer and other cause mortality.

The paper is structured as follows. First, we introduce the illustrative data and we briefly describe the main measure of interest for comparing subgroups, that is the marginal relative survival that is estimated by the standardized relative survival. Then, we investigate the role of a mediator in the exposure–outcome association and explore the impact of interventions that aim to eliminate cancer‐related survival differences. Such interventions can also be quantified using the avoidable deaths measure. Finally, we conclude the paper by summarizing the methods and discussing potential limitations.

## 2 INTRODUCING THE ILLUSTRATIVE EXAMPLE

We demonstrate the methods using an example on survival differences between socioeconomic groups of individuals diagnosed with colon cancer. Data include all individuals diagnosed with colon cancer in England between 2011 and 2013, made available by Public Health England. Available information includes: sex, age and stage at diagnosis as well as deprivation status. Deprivation status is a categorical variable with the deprivation quintiles calculated using the 2010 Index of Multiple Deprivation of the area of patients’ residence at diagnosis (Department for Communities and Local Government, 2010; Neighbourhood Renewal Unit, 2004). Even though deprivation status is a categorical variable with five categories, as the main purpose of the analysis was to demonstrate the measures, we utilize a subset of the population, that is the least and most deprived groups. In England, completeness of stage at diagnosis has improved dramatically after 2012; however, there are a large proportion of missing data for earlier years. As a result, stage at diagnosis was missing for 33.9% of the population. Once again as these data are used only for illustration of the methods, we conducted a complete case analysis including only those with recorded stage at diagnosis. However, our approach can extend to multiple imputation approaches for missing data. The final data included 15,630 patients, 57.6% of which were in the least deprived group. More details on the study population can be found in Table 1.

**TABLE 1:** Number of colon cancer patients (with proportions) for sex, age‐groups and stage at diagnosis by deprivation group

	Deprivation group	
	Least deprived	Most deprived
Sex		
Males	4841 (53.78%)	3500 (52.81%)
Females	4161 (46.22%)	3128 (47.19%)
Age group		
18–44	233 (2.59%)	298 (4.50%)
45–54	537 (5.97%)	470 (7.09%)
55–64	1544 (17.15%)	1267 (19.12%)
65–74	2767 (30.74%)	1877 (28.32%)
75–84	2820 (31.33%)	1970 (29.72%)
85+	1101 (12.22%)	746 (11.25%)
Stage at diagnosis^a^		
I	1338 (14.86%)	912 (13.76%)
II	2644 (29.37%)	1950 (29.42%)
III	2435 (27.05%)	1716 (25.89%)
IV	2585 (28.72%)	2050 (30.93%)

^a^ Stage I‐IV for the least to the most advanced stage at diagnosis.

## 3 MARGINAL RELATIVE SURVIVAL

Let X be a binary variable with X=1for the exposed and X=0 for the unexposed. The conditional all‐cause mortality rate of an individual i, with exposure value $x_{i}\;$ and confounder pattern $z_{i}$, can be partitioned into the conditional expected mortality rate had they not had the cancer,$\;\;h^{*}\left(t|X=x_{i},\;Z_{1}=z_{1i}\right),$ and the conditional excess mortality rate due to cancer, $\lambda (t|X=x_{i},\;Z_{2}=z_{2i})$:

$$h\left(t|X=x_{i},\;Z=z_{i}\right)=h^{*}\left(t|X=x_{i},\;Z_{1}=z_{1i}\right)+\lambda \left(t|X=x_{i},Z_{2}=z_{2i}\right).$$

Z denotes the set of all confounders and consists of subsets$\;\;Z_{1}$ and $Z_{2}$. Subset$\;\;Z_{1}$ denotes the confounders for expected mortality and subset $Z_{2}\;$denotes the confounders for the excess mortality. Often $\;\;Z_{1}$ will be a subset of $Z_{2}$ and in that case $Z_{2}$ will be the equivalent to Z. For instance, sex and age affect other cause mortality but they also affect cancer mortality.

The survival analogue of excess mortality is relative survival. The conditional relative survival, $R(t|X=x_{i},\;Z_{2}=z_{2i}),$ is a function of the conditional all‐cause survival,$\;S(t|X=x_{i},Z=z_{i})$, and the conditional expected survival, $S^{*}\left(t|X=x_{i},\;Z_{1}=z_{1i}\right)$. The conditional all‐cause survival can be written as

(1) $$S\left(t|X=x_{i},Z=z_{i}\right)=S^{*}\left(t|X=x_{i},Z_{1}=z_{1i}\right)R\left(t|X=x_{i},Z_{2}=z_{2i}\right).$$

It is important to point out that there is variation in expected, relative and observed survival between individuals. The expected survival (as well as the expected mortality rates) is typically obtained by available lifetables for the general population that are stratified by confounders $\;Z_{1}$, such as age, sex, calendar year and deprivation status. In a modelling context, relative survival is obtained from a relative survival model in which only confounders $Z_{2}\;$are included in the model. However, the expected survival probabilities of individuals will also be incorporated in this model and these are obtained by the population lifetables which are stratified by confounders $\;\;Z_{1}$.

Relative survival can be interpreted as net survival, that is survival in a hypothetical world where the only possible cause of death is the cancer of interest under the following assumptions: (i) the expected mortality rates that represent mortality due to other causes for the cancer population are appropriate and (ii) the potential times to death from cancer and other causes are conditionally independent (Pavlic & Pohar Perme, 2019). For the first assumption to hold, it is important to include sufficient variables, $Z_{1}$, in the lifetable to ensure comparability between the cancer and the general populations (Pohar Perme et al., 2012; Dickman & Coviello, 2015). The second assumption is the same assumption required in a cause‐specific approach.

Many modelling approaches exist to estimate relative survival and our methods in principle could be applied to all, but in this paper we focus on flexible parametric survival models that use restricted cubic splines to model the baseline log cumulative excess hazard (Cortese & Scheike, 2008; Dickman, Sloggett, Hills, & Hakulinen, 2004; Estève, Benhamou, Croasdale, & Raymond, 1990; Hakulinen & Tenkanen, 1987; Nelson, Lambert, Squire, & Jones, 2007; Royston, 2001). Non‐proportional excess hazards and interactions can easily be incorporated in the model. After fitting the model, a summary of the population prognosis can be obtained as marginal effects such as marginal relative survival functions. We focus on counterfactual marginal relative survival functions within subgroups of the population that are obtained through regression standardization (Sjölander, 2016; Syriopoulou, Rutherford, & Lambert, 2020). Alternative methods to estimate marginal measures like inverse probability weights could also be applied (Cole & Hernán, 2004).

The counterfactual marginal relative survival when setting X=x is defined as

$$\theta \left(t|X=x\right)=E\left[R(t|X=x,Z_{2}\right)],$$

with the expectation taken over $Z_{2}$. To relate the observed and counterfactual outcomes, the assumptions of conditional exchangeability, consistency and positivity need to hold (Hernán & Robins, 2006), both for cancer and other cause mortality. These are discussed in more detail in Syriopoulou et al. (2020). Under these assumptions, the counterfactual marginal relative survival can be estimated as the standardized relative survival. This is given as an average of the predictions of each individual in a study population with N individuals:

$$\hat{\theta }\;\left(t|X\;=\;x\right)=\frac{1}{N}\;\sum_{i\;=\;1}^{N}\hat{R}\left(t|X\;=\;x,\;Z_{2}=\;z_{2i}\right).$$

For the estimation of the counterfactual marginal relative survival, the exposure is set to X=x for every individual in the study population. This is different to utilizing the observed exposure value of individuals, $X\;=x_{i}\;,$ as in Equation (1).

On the mortality scale, the marginal net probability of death can be estimated instead by

$1-\hat{\theta }\left(t|X=x\right)$.

The difference in counterfactual marginal relative survival functions between two different levels of the exposure can also be defined:

(2) $$\theta \left(t|X\;=\;1\right)-\theta \;\left(t|X\;=\;0\right)=\;E\left[R\left(t|X\;=\;1,Z_{2}\right)\right]-E\left[R\left(t|X\;=\;0,Z_{2}\right)\right].$$

This is a comparison of two hypothetical situations: in the first term X is set to 1 for everyone, and in the second term Xis set to 0 for everyone. Under the identifiability assumptions, difference (2) can be estimated by the difference in standardized relative survival functions

$$\frac{1}{N}\sum_{i=1}^{N}\hat{R}\left(t|X=1,Z_{2}=z_{2i}\right)-\frac{1}{N}\sum_{i=1}^{N}\hat{R}\left(t|X=0,Z_{2}=z_{2i}\right).$$

The relative survival difference is a causal effect in the absence of competing risks (Young, Stensrud, Tchetgen Tchetgen, & Hernán, 2020). Difference (2) refers to the cancer‐related difference if everyone was exposed versus if everyone was unexposed, in a hypothetical world where the only possible cause of death is the cancer of interest (Lambert, Dickman, & Rutherford, 2015; Pavlic & Pohar Perme, 2019). All‐cause survival differences, in a setting where other causes of death are present, can also be obtained by incorporating the expected survival:

(3) $$E[S^{*}\left(t|X=1,Z_{1}\right)R\left(t|X=1,Z_{2}\right)]-E[S^{*}\left(t|X=0,Z_{1}\right)R\left(t|X=0,Z_{2}\right)]$$

and is estimated by

$$\begin{array}{c} \frac{1}{N}\sum_{i=1}^{N}S^{*}\left(t|X=1,Z_{1}=z_{1i}\right)\hat{R}\left(t|X=1,Z_{2}=z_{2i}\right),\\ -\frac{1}{N}\sum_{i=1}^{N}S^{*}\left(t|X=0,Z_{1}=z_{1i}\right)\hat{R}\left(t|X=0,Z_{2}=z_{2i}\right). \end{array}$$

In expression (3), survival differences may be due to differences in cancer mortality, other cause mortality or both. More details on marginal estimates and causal effects using relative survival can be found elsewhere (Syriopoulou et al., 2020).

## 4 EXPLORING THE EFFECT OF A MEDIATOR

**FIGURE 1:**
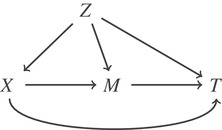
Directed acyclic graph for the relationship of the exposure *X*, time to a specific event *T* and confounding *Z* in the presence of a mediator *M*

Let us assume that we are interested in the potential role of a third variable M, as a mediator, in the association of exposure X and a time‐to‐event outcome T. For instance, Figure 1 demonstrates a setting in which X has both a direct (X→T) and an indirect (X→M→T) effect through M to T (Pearl, 2012). For simplicity, we have assumed the same set of confounders, Z, for X−M and M−T, but this can be generalized to include a different set of confounders for one of them. The setting of Figure 1 can be extended to the relative survival framework. However, within the relative survival framework, we only utilize the information on time to death without knowing the exact cause of death. By extending mediation analysis to relative survival and under certain assumptions, identification of the natural direct and indirect effects is possible. The mediators that will be considered here are assumed to affect only cancer mortality rates and have no effect on other cause mortality rates.

Let $M^{x}$ 
denote the counterfactual mediator distribution when intervening to set the exposure X to level x. Let $R(t|X=y,Z_{2},\;M^{x}$) be the counterfactual relative survival function when intervening to set the exposure X to level y and the mediator M to $M^{x}$. Level y is set to values 0 and 1 for the unexposed an exposed, respectively. Sometimes it is possible to have x=y, that is the relative survival that would have been observed if both the exposure and the counterfactual mediator distribution have been set to the same exposure level.

Within the relative survival framework, the natural direct effect ($\mathrm{ND}E_{RS}$) is defined as the difference in marginal relative survival between the exposed and unexposed if both groups had the same mediator distribution as the unexposed (setting M to $M^{0}$ and thus M remains the same for each patient in both terms):

(4) $$\mathrm{ND}E_{RS}=E\left[R\left(t|X=1,Z_{2},\;M^{0}\right)]-E[R\left(t|X=0,Z_{2},\;M^{0}\right)\right].$$

The natural indirect effect ($\mathrm{NI}E_{RS}$), which gives the effect of the mediator, is defined as the difference when setting X=1 and comparing the effects of having their own mediator distribution (setting M to $M^{1})$ versus if they had the same mediator distribution as the unexposed (setting M to $M^{0}$):

(5) $$\mathrm{NI}E_{RS}=E\left[R\left(t|X=1,Z_{2},\;M^{1}\right)]-E[R\left(t|X=1,Z_{2},\;M^{0}\right)\right]\;.$$

In (5), X is allowed to influence relative survival only through its influence on M.

Identification of $\mathrm{ND}E_{RS}$ and $\mathrm{NI}E_{RS}$ is possible under standard mediation analysis assumptions that are now extended to both outcomes, that is cancer and other causes (De Stavola, et al., 2014; Pearl, 2001). For the rest of this paragraph, referring to the outcome will imply both cancer and other causes. First, no interference assumption states that a patient's exposure has no effect on the outcome of another (both cancer and other cause) and that a patient's mediator value does not influence the outcome of another patient. In addition, an individual's exposure has no effect on the mediator of another individual. Second, consistency states that an individual's outcome under the actual values of X=x and M=m is equal to the outcome that would be observed under an intervention of setting the exposure X=x and the mediator to M=m. Consistency also expands so that (i) the outcome under the actual value of X=x is equal to the outcome that would be observed under an intervention of setting X=x and $M=M^{x}$ as well as (ii) $M^{x}=M$ when the actual value is X=x. Finally, conditional exchangeability states that there is (i) no unmeasured exposure–outcome confounding conditionally on confounders, (ii) no unmeasured mediator–outcome confounding conditionally on exposure and confounders, (iii) no unmeasured exposure–mediator confounding conditional on confounders and (iv) no mediator–outcome confounder affected by exposure. Achieving conditional exchangeability for other cause mortality depends on the level of stratification in the lifetables that are used to incorporate expected mortality rates. If the variables of the lifetables are insufficient then this assumption is violated. Various methods have been suggested to incorporate factors that are not available on population level (Bower et al., 2017; Ellis, Coleman, & Rachet, 2014; Rubio, Rachet, Giorgi, Maringe, & Belot, 2019).

The $\mathrm{ND}E_{RS}$ and $\mathrm{NI}E_{RS}$ can be estimated by (Pearl, 2001; Pearl, 2012)

$$\begin{array}{ccc} \hat{\mathrm{ND}E_{RS}} & = & \frac{1}{N}\sum_{i=1}^{N}\sum_{m}\hat{R}\left(t|X=1,\;Z_{2}=z_{2i},\;M=m\right)\hat{\;P}\left(M=m|X=0,\;Z_{2}=z_{2i}\right)\\ & & -\frac{1}{N}\sum_{i=1}^{N}\sum_{m}\hat{R}\left(t|X=0,\;Z_{2}=z_{2i},\;M=m\right)\hat{\;P}\left(M=m|X=0,\;Z_{2}=z_{2i}\right) \end{array}$$

$$\begin{array}{ccc} \hat{\mathrm{NI}E_{RS}} & = & \frac{1}{N}\sum_{i=1}^{N}\sum_{m}\hat{R}\left(t|X=1,\;Z_{2}=z_{2i},\;M=m\right)\hat{\;P}\left(M=m|X=1,\;Z_{2}=z_{2i}\right)\\ & & -\frac{1}{N}\sum_{i=1}^{N}\sum_{m}\hat{R}\left(t|X=1,\;Z_{2}=z_{2i},\;M=m\right)\hat{\;P}\left(M=m|X=0,\;Z_{2}=z_{2i}\right) \end{array}$$

with m taking values 0 and 1 for a binary mediator M. For a mediator with more levels, the summation is taken over all levels. Also, $\hat{\;P}\left(M=m|X=x,\;Z_{2}=z_{2i}\right)$ is the estimated probability of being in a specific level of the mediator given exposure and confounders.

Detailed steps on the estimation can be found in Box 1. To account for the uncertainty on the probabilities estimated in Step 3 and the survival functions of Step 4, bootstrap‐based standard errors are obtained. By performing parametric bootstrap, the parameters are drawn repeatedly from a multivariate normal distribution and for each draw we obtain both estimates and the variance covariance matrix that are finally combined (Efron &Tibshirani, 1993). An example of Stata code for obtaining predictions can be found in the Supporting Information.

The proportion of the total causal effect ($\mathrm{TC}E_{RS}$) that is due to the mediator within the net survival setting is then defined as

$$PM_{RS}=\frac{\mathrm{NI}E_{RS}}{\mathrm{TC}E_{RS}},$$

with the $\mathrm{TC}E_{RS}=\mathrm{ND}E_{RS}+\mathrm{NI}E_{RS}.$

The $\mathrm{ND}E_{RS}$ and $\mathrm{NI}E_{RS}$ refer to differences in relative survival between exposure groups in a net‐survival setting where the only possible cause of death is the cancer of interest and therefore yield differences that exist only due to the cancer of interest and not other causes.

Instead of focusing on the net survival setting as in (4) and (5), it is also possible to obtain estimates in a situation where both cancer and other causes are present. This can be done by incorporating the expected survival and forming contrasts of all‐cause survival. There are many ways to incorporate the expected survival. For instance, the following contrasts can be formed:

$$\mathrm{ND}E_{\mathrm{AC}1}=E\left[S^{*}\left(t|X=1,Z_{1}\right)R\left(t|X=1,Z_{2},\;M^{0}\right)\right]-E\left[S^{*}\left(t|X=0,Z_{1}\right)R\left(t|X=0,Z_{2},\;M^{0}\right)\right]$$

$$\mathrm{NI}E_{\mathrm{AC}1}=E\left[S^{*}\left(t|X=1,Z_{1}\right)R\left(t|X=1,Z_{2},\;M^{1}\right)\right]-E\left[S^{*}\left(t|X=1,Z_{1}\right)R\left(t|X=1,Z_{2},\;M^{0}\right)\right].$$

For the $\mathrm{ND}E_{\mathrm{AC}1},$ the first term includes the expected survival if exposure was set to 1 for everyone, $S^{*}\left(t|X=1,Z_{1}\right)$, and the second term includes the expected survival by setting X=0, $S^{*}\left(t|X=0,Z_{1}\right)$. Therefore, for $\mathrm{ND}E_{\mathrm{AC}1}$, survival differences may be due to differential cancer mortality, other cause mortality or both. Understanding the mechanisms of all‐cause survival differences can be complex. Thus, it might be of interest to obtain the following measures instead:

$\mathrm{ND}E_{\mathrm{AC}2}=E\left[S^{*}\left(t|X,Z_{1}\right)R\left(t|X=1,Z_{2},\;M^{0}\right)\right]-E\left[S^{*}\left(t|X,Z_{1}\right)R\left(t|X=0,Z_{2},\;M^{0}\right)\right]$

$\mathrm{NI}E_{\mathrm{AC}2}=\;E\left[S^{*}\left(t|X,Z_{1}\right)R\left(t|X=1,Z_{2},\;M^{1}\right)\right]-E\left[S^{*}\left(t|X,Z_{1}\right)R\left(t|X=1,Z_{2},\;M^{0}\right)\right].$

For $\mathrm{ND}E_{\mathrm{AC}2}$ and $\mathrm{NI}E_{\mathrm{AC}2}$, survival differences can only be due to the cancer of interest as the expected survival, $S^{*}\left(t|X,Z_{1}\right),$ is incorporated using the observed distribution of the exposure for both contrasting terms. This is the key difference with $\mathrm{ND}E_{\mathrm{AC}1}$ to which the expected survival was incorporated by setting X=1 or X=0 for everyone in the study population.

The direct and the indirect effects can also be obtained within subsets of the whole population. For instance, the NDE among the exposed could be estimated by standardizing only to patients of the exposed group, $N_{X=1}$. Such contrasts can be useful for assessing the potential impact of interventions that aim to eliminate differences between groups by either focusing on the mediator distribution or the cancer‐related survival (i.e. relative survival).

It is important to note that the interventions considered in this paper are assumed to have no impact on other cause mortality rates (i.e. other cause mortality remains unchanged after such interventions). The two competing events are assumed to be conditionally independent. Even when referring to an all‐cause setting, it is assumed that any potential impact of the intervention on survival is due to changes in cancer mortality. For $\mathrm{ND}E_{\mathrm{AC}1}$, some of the survival differences will be due to differences in other cause mortality but this would be a result of differential background mortality between exposed and unexposed and not a result of the intervention. The motivation for this distinction is that the effect of certain interventions would be separable across the two competing causes; for instance, increased cancer screening engagement would likely impact only on the mortality for the cancer cause specifically, whilst only indirectly impacting on other‐cause death probabilities but not the underlying other‐cause mortality rates.

### 4.1 Example

We explored survival differences following a diagnosis of colon cancer between socioeconomic groups by fitting a flexible parametric survival model with 5 degrees of freedom for the baseline excess hazard including sex, deprivation status, age and stage at diagnosis and allowing for time‐dependent effects for deprivation, age and stage (3 degrees of freedom). Age at diagnosis was included in the model as a continuous non‐linear variable using restricted cubic splines with 3 degrees of freedom. An interaction between stage and deprivation was also allowed. A multinomial regression model was fitted for stage including age as a continuous non‐linear variable, deprivation status and sex. The 95% confidence intervals were obtained using the standard deviation of a parametric bootstrap sample with k=500.

**FIGURE 2:**
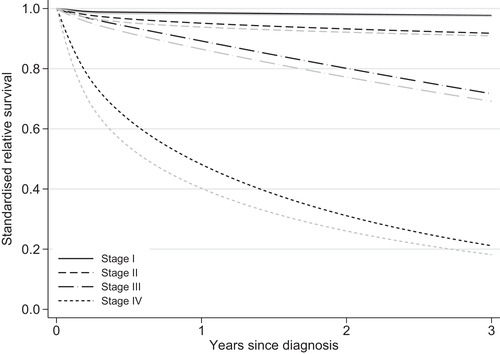
Standardized estimates of relative survival by years since diagnosis and stage at diagnosis. Black and grey lines refer to the relative survival of least and most deprived patients, respectively

**FIGURE 3:**
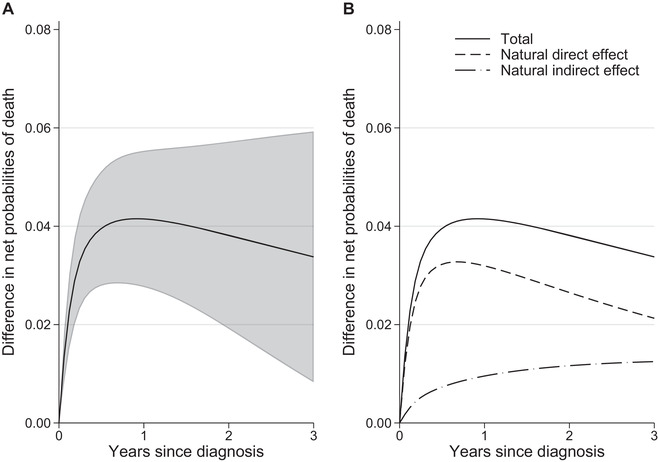
(A) Total causal effect, defined as the difference in standardized net probabilities of death, with 95% confidence intervals and (B) partitioning of the total causal effect to the natural direct and indirect effect due to stage at diagnosis

**FIGURE 4:**
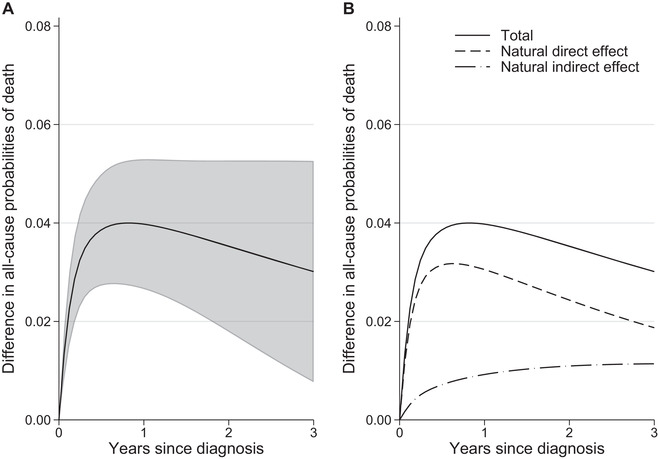
(A) Total causal effect, defined as the difference in standardized all cause probabilities of death, with 95% confidence intervals and (B) partitioning of the total causal effect to the natural direct and indirect effect due to stage at diagnosis

We found differences in standardized relative survival between the least and most deprived groups, especially for the most advanced stages (Figure 2). Differences were also observed in the stage distribution, as a higher proportion of the most deprived were diagnosed in a more advanced stage (Table 1). The role of stage as a potential mediator in the association of deprivation and survival time was further investigated by obtaining the $\hat{\mathrm{ND}E_{RS}}$ and $\hat{\mathrm{NI}E_{RS}}$ that are due to stage specific survival differences and due to the stage differences, respectively, in the net survival setting where the only possible cause of death is colon cancer (Figure 3). Three years after diagnosis a total difference of 3.38% (95% CI: [0.83, 5.93]) was observed in standardized net probabilities of death and 1.25% (95% CI: [0.22, 2.28]) of the difference was attributed to differences in stage at diagnosis. As a result, the proportion of total differences in standardized net probability of death that was mediated through stage at 3 years was 37% (i.e. 1.25/1.253.383.38). To obtain an estimate of cancer‐related differences in the all‐cause setting, where other causes of death are present, the $\hat{\mathrm{ND}E_{AC2}}$ and $\hat{\mathrm{NI}E_{AC2}}$ were also estimated (Figure 4). We found that the indirect effect due to stage was 1.14% (95% CI: [0.24, 2.04]) and the total difference in probabilities of death was equal to 3.01% (95% CI: [0.77, 5.26]). Thus, 38% of the difference in this all‐cause setting was mediated through stage.

## 5 AVOIDABLE DEATHS UNDER HYPOTHETICAL INTERVENTIONS

The impact of eliminating differences between groups, in the presence of both cancer and other cause mortality, can also be quantified as the avoidable deaths after an intervention (Syriopoulou et al., 2020). Such an intervention would be to eliminate differences in the mediator distribution. As mentioned earlier, the mediators considered in this paper are assumed to affect only the cancer mortality rates and have no effect on the rates of other cause mortality.

The avoidable deaths is a time‐specific measure and has the interpretation of postponable deaths as eventually all deaths will be realized. Although the avoidable deaths can also be defined for the whole population, here we focus on the avoidable deaths among the exposed, that is obtaining marginal estimates using a subset of the population.

Assume that we are interested in the avoidable deaths under an intervention that aims to eliminate differences in the distribution of the mediator between exposed and unexposed, while keeping other cause mortality rates unchanged. First, we need the number of deaths for the exposed that is given by multiplying the number of exposed patients diagnosed in a typical calendar year, $N^{*}$ with the probability of death:

(6) $$D_{1}\left(t|X=1,\;M^{1}\right)=N^{*}\left(1-E\left[S^{*}\left(t|X=1,Z_{1}^{X=1}\right)R\left(t|X=1,Z_{2}^{X=1},\;M^{1}\right)\right]\right),$$

with $Z_{1}^{X=1}$and $Z_{2}^{X=1}$denoting the covariates for the exposed, for the expected and relative survival, respectively.

Then, the expected number of deaths under the intervention can be derived by shifting the mediator distribution of the exposed to the one of the unexposed (setting M to $M^{0}$):

(7) $$D_{M}\left(t|X=1,\;M^{0}\right)=N^{*}\left(1-E\left[S^{*}\left(t|X=1,Z_{1}^{X=1}\right)R\left(t|X=1,Z_{2}^{X=1},\;M^{0}\right)\right]\right).$$

The avoidable deaths from eliminating differences in the mediator distribution is given by

$AD_{RS}=D_{1}\left(t|X=1,\;M^{1}\right)-D_{M}\left(t|X=1,\;M^{0}\right)$.

For the identification of $AD_{RS}$, assumptions similar to the one described for of $\mathrm{ND}E_{RS}$ and $\mathrm{NI}E_{RS}$ are required.

It is important to note that in the above scenario, we keep the expected survival of the exposed group unchanged and we assess an intervention that aims to shift the mediator distribution of the exposed to that of the unexposed with no impact on other cause mortality rates. As a result, the potential impact of the interventions considered here will only be due to changes in cancer mortality rates.

A key point for the interpretation of the avoidable deaths is the number of patients $N^{*}$ applied in (6) and (7). $N^{*}$ can be any number relevant to the study population. For instance, it could be the number of exposed patients diagnosed in the most recent year in our data, or it could be derived by adding all exposed patients diagnosed during the total duration of the follow‐up divided by the number of years available. Some might also consider calculating the avoidable deaths per 1000 patients. When interpreting the results, it is important to keep in mind potential differences between the population being marginalized over, which in the above example is all the exposed patients of the study (over a range of calendar years), and the population used for $N^{*}$. In extreme cases, the covariate pattern might have changed over calendar time suggesting that a choice must be made over the most relevant information to present. It may be preferable to marginalize over a specific restricted population, with the appropriately calculated N* for that population.

### 5.1 Example

We estimated the avoidable deaths under two hypothetical interventions:

1. ‘eliminating differences in the *stage at diagnosis distribution* as well as *relative survival differences* between the least and most deprived groups’ (scenario 1).
2. ‘eliminating differences in the *stage at diagnosis distribution* between the least and most deprived groups’ (scenario 2).

**FIGURE 5:**
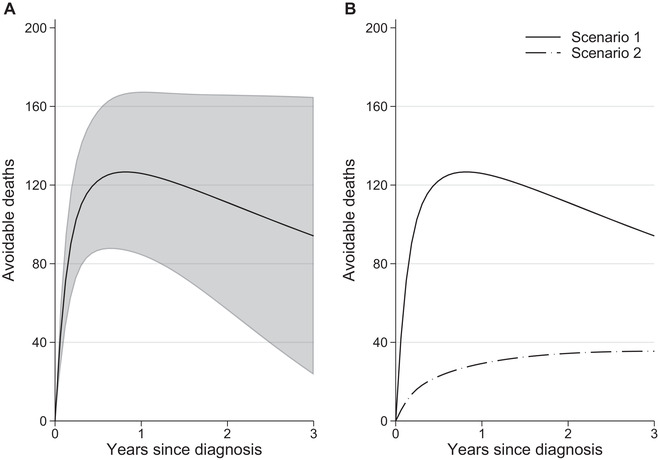
(A) Total avoidable deaths by removing relative survival and stage differences between the least and most deprived groups (Scenario 1) with 95% confidence intervals and (B) partitioning total avoidable deaths to those under an intervention of “eliminating differences in the stage at diagnosis distribution” (Scenario 2)

For scenario 1, we shifted the relative survival and stage at diagnosis distribution of the most deprived patients to that of the least deprived, that is the most advantaged group. For scenario 2, we shifted the stage at diagnosis distribution of the most deprived to that of the least deprived group. For both scenarios, we kept the expected survival of the most deprived unchanged. Three years after diagnosis 94 (95% CI: [24, 165]) avoidable deaths would be observed in total, out of 3228 ($i.e.\;\;N^{*})$ patients from the most deprived group diagnosed in 2013 the most recent year in our cohort study. Partitioning that further, we found that 35 (95% CI: [7, 64]) deaths of the total deaths would be from eliminating stage differences (scenario 2) and the remaining 59 would be from removing relative survival differences (Figure 5).

## 6 DISCUSSION

We extended mediation analysis to the relative survival framework as a tool for investigating differences in cancer prognosis. Using relative survival enables forming contrasts of either relative survival in which the only possible cause of death is the cancer of interest or in all‐cause survival in which both the cancer and other causes are present. We showed that, if certain assumptions hold, mediation analysis can be applied to identify the indirect effect due to a mediator both in the net survival and all‐cause setting. For further exploration of the survival differences, the potential impact of interventions can be estimated as the number of deaths that could be avoided within a time frame. The interventions considered here focus on cancer‐related mortality rates and are assumed to have no impact on other cause mortality rates. Because we are in a competing risks setting, changing the cancer mortality rates will increase the probability of dying from other causes, even if the other cause mortality rates remain unchanged.

To account for the uncertainty in the probability weights and the predictions of standardized survival (Step 3 and Step 4 of Box 1), we obtain bootstrap‐based standard errors by performing parametric bootstrap for the parameter estimates of both models. Confidence intervals are obtained using the relevant quantiles of the bootstrap samples estimates distribution or their standard deviation. Alternative approaches could be applied such as M‐estimation methods that would shorten the computational time (Stefanski & Boos, 2002).

**BOX 1: Algorithm for obtaining the natural direct and indirect effects**

Step 1. Fit a parametric relative survival model for the time‐to event outcome including the exposure, mediator, potential confounders and appropriate interactions and time‐dependent effects.

Step 2. Fit a model for the mediator including the exposure and confounders. For example, for a binary mediator this could be a logistic regression model and for a mediator with more categories this could be a multinomial regression model.

Step 3. For each individual in the study population obtain predictions for the probability of being in a specific level of the mediator, $\hat{P}\left(M=m|X=x,\;Z_{2}=z_{2i}\right)$, at each level of the exposure X=x.

Step 4. Obtain predictions of the standardized relative survival functions at each level of X=x, as a weighted average of the individual relative survival functions $\hat{R}\left(t|X=x,\;Z_{2}=z_{2i},M=m\right)$, using the predictions of Step 3 as weights. Contrasts of these predictions can be formed to obtain the $\hat{\mathrm{ND}E_{RS}}$ and $\hat{\mathrm{NI}E_{RS}}$.

Step 5. Repeat from Step 3 for k times while performing parametric bootstrap for the parameter estimates for both models.

Step 6. Calculate 95% confidence intervals either by taking the 2.5% and 97.5% quantiles of the $\hat{\mathrm{ND}E_{RS}}$ and $\hat{\mathrm{NI}E_{RS}}$ estimates across the bootstrapped samples or by using the standard deviation of the estimates obtained from the bootstrap samples.

Identifying interventions of eliminating all‐cause differences can be challenging. This is because differences in all‐cause survival can be the result of complex mechanisms that involve both cancer‐related and other cause differences. In this paper, we utilized relative survival and focus on cancer‐related differences. Quantifying differences in a real‐world setting having focused on eliminating cancer‐related differences alone is also possible by incorporating the expected survival probabilities. An intervention that aims to eliminate cancer‐related differences without intervening on the other‐cause mortality might be easier to identify. However, one could argue that our intervention is still not well defined (Hernán & Robins, 2006). Changing the cancer mortality of one exposure group while keeping the others the same might not be straightforward in practice. For instance, an intervention that aims to increase cancer awareness in the most deprived patents will most probably increase awareness also in the least deprived group. If this is the case, our estimates will provide a lower bound of the actual population benefit of the intervention. Nevertheless, quantifying the impact of such a conceptual intervention in a formalized causal framework gives a firm basis to improve our understanding on cancer disparities even if such an intervention is difficult to identify in practice (Glymour & Spiegelman, 2017; Krieger & Davey Smith, 2016; Pearl, 2018; Vandenbroucke, 2016).

Interpretation as causal effects depends on the validity of standard mediation analysis assumptions that are now extended to the relative survival framework and therefore need to hold for both outcomes, cancer and other cause survival times. These are no interference, consistency and conditional exchangeability (De Stavola et al. 2014; Pearl, 2001). Achieving conditional exchangeability for the other cause mortality depends on the availability of relevant lifetables that are used to represent the other cause mortality of the cancer population. Causal interpretation is only appropriate when lifetables are sufficiently stratified, but in principle lifetables can be constructed for any number of factors. To deal with this issue and consider other risk factors that are not always available on a population level, adjustments at the expected mortality rates have been suggested (Bower et al., 2017; Ellis et al., 2014; Rubio et al., 2019). Finally, we have assumed no intermediate confounders, that is no mediator–outcome confounder affected by the exposure (cross‐world independence assumption). Methods that do not require the cross‐world assumption have been suggested before by either using a weighting‐based approach with the limitation of not adding to the total effect or a Monte Carlo–based regression approach that applies also to multiple mediators (VanderWeele, Vansteelandt & Robins, 2014; Vansteelandt & Daniel, 2017). In principle, our methods can be extended to settings with intermediate confounders and this consists part of future work.

Further assumptions that relate to relative survival should also hold: appropriate expected mortality rates and conditional independence of the outcomes. The former highlights the importance of representative lifetables, and the latter requires that relative survival and expected survival are independent after adjusting for sufficient variables (Lambert et al., 2015; Seppä, Hakulinen, Läärä, & Pitkäniemi, 2016). Under these assumptions, relative survival can be interpreted as a net survival measure in a hypothetical world with cancer being the only possible cause of death. If interest is in obtaining “real”‐world probabilities, we can estimate measures such as standardized crude probabilities and avoidable deaths measures, by incorporating expected mortality rates.

The exposures and confounders considered in this paper are time‐fixed. However, appropriate methodology that accounts for time‐varying exposures or confounders has been suggested before and this can be extended in the relative survival framework to allow the estimation of relevant causal parameters (Robins, Hernán, & Brumback, 2000).

Even though cancer inequalities had been well documented, understanding the underlying determinants of these differences is a challenging task. In this paper, we utilized mediation analysis methods and incorporated the relative survival framework to address these challenges. The proposed method has the advantage of allowing us to focus on cancer‐related differences, the underlying determinants of which may be easier to identify in comparison with all‐cause differences. Adjusting for sufficient confounders is essential, and caution is required when interpreting the findings.

## CONFLICT OF INTEREST

The authors have declared no conflict of interest.

### OPEN RESEARCH BADGES

This article has earned an Open Data badge for making publicly available the digitally‐shareable data necessary to reproduce the reported results. The data is available in the Supporting Information section.

This article has earned an open data badge “**Reproducible Research**” for making publicly available the code necessary to reproduce the reported results. The results reported in this article could fully be reproduced.

## Supporting information



Supporting Information

Supporting Information
