# Supplementary material for: Understanding disparities in cancer prognosis: An extension of mediation analysis to the relative survival framework
Source: Biom J. 2020 Dec 14;63(2):341–53. doi: 10.1002/bimj.201900355 (PMC7898837; doi:10.1002/bimj.201900355)
Supplement: Supplementary file 1 — Supporting Information [file BIMJ-63-341-s002.docx]

Supplementary material

Stata code

More information on how to obtain predictions for the natural direct and indirect effect in Stata are given below, following the steps of Box 1 of the paper.

Step 1. Fit a parametric relative survival model for the time-to event outcome including the exposure, mediator, potential confounders and appropriate interactions and time-dependent effects. In our example, we fitted a flexible parametric model that uses restricted cubic splines to model the baseline excess hazard. For simplicity, here we assume that we are interested in a flexible parametric model with 3 df for the baseline hazard and 2 df for the time-dependent effects. More specifically, the model includes deprivation status (dep5: equal to 1 for the most deprived patients and 0 the least deprived), age (rcsa1 rcsa2 rcsa3: continuous, non linear variable with 3 splines), gender (gender: 1 for females and 0 for males) and stage at diagnosis (stage1 stage2 stage3 stage4: with stage1 the reference category). This model can be fitted in Stata after declaring the data (stset) as survival data and merging in the expected mortality rates as:

stpm2 dep5 rcsa1 rcsa2 rcsa3 gender stage2 stage3 stage4, df(3) ///

tvc(rcsa1 rcsa2 rcsa3 dep5 stage2 stage3 stage4) dftvc(2) ///

scale(h) bhaz(rate)

//Store the model parameters

estimates store surv

In the above model we assumed no interactions for simplicity but this can easily be included in the model. The bhaz(rate)option is applied to denote that this is a relative survival model with rate being the expected mortality rates variable.

Step 2. Fit a model for the mediator including the exposure and confounders. For example, for a binary mediator this could be a logistic regression model and for a mediator with more categories this could be a multinomial regression model. Here cancer_stage indicates the mediator variable with levels 1,2,3,4.

//Fit a multinomial regression model for the most deprived

mlogit cancer_stage rcsa1 rcsa2 rcsa3 gender if dep5==1

//Store the model parameters

estimates store ph1

//Fit a multinomial regression model for the least deprived

mlogit cancer_stage rcsa1 rcsa2 rcsa3 gender if dep5==0

//Store the model parameters

estimates store ph0

Step 3. For each individual in the study population obtain predictions for the probability of being in a specific level of the mediator, $\hat{P}\left( M=m | X=x, {Z_{2}=z}_{2i} \right)$, at each level of the exposure $X=x$. For the confidence intervals, first draw the parameters from a multivariate normal distribution:

//For the least deprived (dep5=0)

preserve

estimates restore ph0

matrix b0 = e(b)

matrix V0= e(V)

drawnorm b1_rcsa1 b1_rcsa2 b1_rcsa3 b1_gender b1_cons ///

b2_rcsa1 b2_rcsa2 b2_rcsa3 b2_gender b2_cons ///

b3_rcsa1 b3_rcsa2 b3_rcsa3 b3_gender b3_cons ///

b4_rcsa1 b4_rcsa2 b4_rcsa3 b4_gender b4_cons, mean(b0) cov(V0)

n(1) clear

list

local cnames: colfullnames b0

local rnames: rowfullnames b0

mkmat b1_rcsa1 b1_rcsa2 b1_rcsa3 b1_gender b1_cons ///

b2_rcsa1 b2_rcsa2 b2_rcsa3 b2_gender b2_cons ///

b3_rcsa1 b3_rcsa2 b3_rcsa3 b3_gender b3_cons ///

b4_rcsa1 b4_rcsa2 b4_rcsa3 b4_gender b4_cons, matrix(b0_tmp)

matrix colnames b0_tmp = `cnames'

matrix rownames b0_tmp = `rnames'

erepost b = b0_tmp V=V0, noesample

restore

//Obtain predictions for stages 1,2,3 and 4

predict p01 p02 p03 p04

//Similarly obtain predictions, p11 p12 p13 p14, for the most deprived group (dep5=1)

Step 4. Obtain predictions of the standardized relative survival functions at each level of $X=x$, $\hat{R}\left( t | X=x, {Z_{2}=z}_{2i}, M=m \right),$ using the predictions of Step 3 as weights. Contrasts of these predictions can be formed to obtain the $\hat{\mathrm{ND}E_{RS}}$ and $\hat{\mathrm{NI}E_{RS}}$. Once again, in order to obtain the confidence intervals, first draw the model parameters from a multivariate normal distribution. This is done in a similar way as before:

preserve

estimates restore surv

matrix bsurv = e(b)

matrix V3surv= e(V)

drawnorm b_dep5 b_rcsa1 b_rcsa2 b_rcsa3 b_gender b_stage2 b_stage3

b_stage4 ///

b_rcs1 b_rcs2 b_rcs3 ///

b_rcs_rcsa11 b_rcs_rcsa12 b_rcs_rcsa21 b_rcs_rcsa22

b_rcs_rcsa31 b_rcs_rcsa32 ///

b_rcs_dep51 b_rcs_dep52 ///

b_rcs_stage21 b_rcs_stage22 b_rcs_stage31 b_rcs_stage32

b_rcs_stage41 b_rcs_stage42 ///

b_cons ///

b_d_rcs1 b_d_rcs2 b_d_rcs3 ///

b_d_rcs_rcsa11 b_d_rcs_rcsa12 b_d_rcs_rcsa21

b_d_rcs_rcsa22 b_d_rcs_rcsa31 b_d_rcs_rcsa32 ///

b_d_rcs_dep51 b_d_rcs_dep52 ///

b_d_rcs_stage21 b_d_rcs_stage22 b_d_rcs_stage31

b_d_rcs_stage32 b_d_rcs_stage41 b_d_rcs_stage42 ,

mean(bsurv) cov(V3surv) n(1) clear

list

local cnames: colfullnames bsurv

local rnames: rowfullnames bsurv

mkmat b_dep5 b_rcsa1 b_rcsa2 b_rcsa3 b_gender b_stage2 b_stage3

b_stage4 ///

b_rcs1 b_rcs2 b_rcs3 ///

b_rcs_rcsa11 b_rcs_rcsa12 b_rcs_rcsa21 b_rcs_rcsa22

b_rcs_rcsa31 b_rcs_rcsa32 ///

b_rcs_dep51 b_rcs_dep52 ///

b_rcs_stage21 b_rcs_stage22 b_rcs_stage31 b_rcs_stage32

b_rcs_stage41 b_rcs_stage42 ///

b_cons ///

b_d_rcs1 b_d_rcs2 b_d_rcs3 ///

b_d_rcs_rcsa11 b_d_rcs_rcsa12 b_d_rcs_rcsa21

b_d_rcs_rcsa22 b_d_rcs_rcsa31 b_d_rcs_rcsa32 ///

b_d_rcs_dep51 b_d_rcs_dep52 ///

b_d_rcs_stage21 b_d_rcs_stage22 b_d_rcs_stage31

b_d_rcs_stage32 b_d_rcs_stage41 b_d_rcs_stage42 ,

matrix(bsurv_tmp)

matrix colnames bsurv_tmp = `cnames'

matrix rownames bsurv_tmp = `rnames'

erepost b = bsurv_tmp V=V3surv, noesample

restore

The b_rcs1 b_rcs2 b_rcs3 denote the model parameters for the 3 splines used to model the baseline excess hazard and b_d_rcs1 b_d_rcs2 b_d_rcs3 denote the derivatives of these splines. Please note that in flexible parametric models, the splines and their derivatives are part of the model parameters and therefore should be included in the draw.

Prediction for the $\hat{\mathrm{ND}E_{RS}}$ and $\hat{\mathrm{NI}E_{RS}}$ can be obtained using standsurv command and specifying the at options to form the contrasts of interest:

//For the NDE we compare the most deprived with the least deprived while setting M to M0 for everyone.

//Here each at option refers to a specific level of the exposure and a specific stage at diagnosis. All the at options are then combined to form the contrast of interest, indicated by the lincom() option.

//In this example, 8 at options are used. This is because there are 2 deprivation groups with 4 stage at diagnosis each.

//The option atindweights() is used to set the mediator distribution to that of the unexposed group. This is done, by applying the weights of Step 3.

standsurv, failure timevar(timevar) ///

at1(dep5 1 stage2 0 stage3 0 stage4 0 stage2dep5 0 stage3dep5 0

stage4dep5 0, atindweights(p01)) ///

at2(dep5 1 stage2 1 stage3 0 stage4 0 stage2dep5 1 stage3dep5 0

stage4dep5 0, atindweights(p02)) ///

at3(dep5 1 stage2 0 stage3 1 stage4 0 stage2dep5 0 stage3dep5 1

stage4dep5 0, atindweights(p03)) ///

at4(dep5 1 stage2 0 stage3 0 stage4 1 stage2dep5 0 stage3dep5 0

stage4dep5 1, atindweights(p04)) ///

at5(dep5 0 stage2 0 stage3 0 stage4 0 stage2dep5 0 stage3dep5 0

stage4dep5 0, atindweights(p01')) ///

at6(dep5 0 stage2 1 stage3 0 stage4 0 stage2dep5 0 stage3dep5 0

stage4dep5 0, atindweights(p02)) ///

at7(dep5 0 stage2 0 stage3 1 stage4 0 stage2dep5 0 stage3dep5 0

stage4dep5 0, atindweights(p03)) ///

at8(dep5 0 stage2 0 stage3 0 stage4 1 stage2dep5 0 stage3dep5 0

stage4dep5 0, atindweights(p04)) ///

lincom(1 1 1 1 -1 -1 -1 -1) lincomvar(tde)

//For the NIE we set everyone to have dep5==1 and form a contrast if they has the M1 versus if they had M0.

//The option atindweights() is used to set the mediator distribution M1 and M0.

standsurv, failure timevar(timevar) ///

at1(dep5 1 stage2 0 stage3 0 stage4 0 stage2dep5 0 stage3dep5 0

stage4dep5 0, atindweights(p11)) ///

at2(dep5 1 stage2 1 stage3 0 stage4 0 stage2dep5 1 stage3dep5 0

stage4dep5 0, atindweights(p12)) ///

at3(dep5 1 stage2 0 stage3 1 stage4 0 stage2dep5 0 stage3dep5 1

stage4dep5 0, atindweights(p13)) ///

at4(dep5 1 stage2 0 stage3 0 stage4 1 stage2dep5 0 stage3dep5 0

stage4dep5 1, atindweights(p14)) ///

at5(dep5 1 stage2 0 stage3 0 stage4 0 stage2dep5 0 stage3dep5 0

stage4dep5 0, atindweights(p01)) ///

at6(dep5 1 stage2 1 stage3 0 stage4 0 stage2dep5 1 stage3dep5 0

stage4dep5 0, atindweights(p02)) ///

at7(dep5 1 stage2 0 stage3 1 stage4 0 stage2dep5 0 stage3dep5 1

stage4dep5 0, atindweights(p03)) ///

at8(dep5 1 stage2 0 stage3 0 stage4 1 stage2dep5 0 stage3dep5 0

stage4dep5 1, atindweights(p04)) ///

lincom(1 1 1 1 -1 -1 -1 -1) lincomvar(tie)

Step 5. For the confidence intervals, repeat from Step 3 for $k$ times while performing parametric bootstrap for the parameter estimates for both models.

Step 6. Calculate 95% confidence intervals either by taking the 2.5% and 97.5% quantiles of the $\hat{\mathrm{ND}E_{RS}}$ and $\hat{\mathrm{NI}E_{RS}}$ estimates across the bootstrapped samples or by using the standard deviation of the estimates obtained from the bootstrap samples.

Predictions in an all-cause setting, $\hat{\mathrm{ND}E_{AC2}}$ and $\hat{\mathrm{NI}E_{AC2}}$ , are obtained by incorporating the expected mortality in the contrasts of Step 4. This is done in standsurv using the option expsurv(). For example, the $\hat{\mathrm{NI}E_{AC2}}$ is given by

standsurv, failure timevar(timevar) ///

at1(dep5 1 stage2 0 stage3 0 stage4 0 stage2dep5 0 stage3dep5 0

stage4dep5 0, atif(dep5==1) atindweights(p11)) ///

at2(dep5 1 stage2 1 stage3 0 stage4 0 stage2dep5 1 stage3dep5 0

stage4dep5 0, atif(dep5==1) atindweights(p12)) ///

at3(dep5 1 stage2 0 stage3 1 stage4 0 stage2dep5 0 stage3dep5 1

stage4dep5 0, atif(dep5==1) atindweights(p13)) ///

at4(dep5 1 stage2 0 stage3 0 stage4 1 stage2dep5 0 stage3dep5 0

stage4dep5 1, atif(dep5==1) atindweights(p14)) ///

at5(dep5 1 stage2 0 stage3 0 stage4 0 stage2dep5 0 stage3dep5 0

stage4dep5 0, atif(dep5==1) atindweights(p01)) ///

at6(dep5 1 stage2 1 stage3 0 stage4 0 stage2dep5 1 stage3dep5 0

stage4dep5 0, atif(dep5==1) atindweights(p02)) ///

at7(dep5 1 stage2 0 stage3 1 stage4 0 stage2dep5 0 stage3dep5 1

stage4dep5 0, atif(dep5==1) atindweights(p03)) ///

at8(dep5 1 stage2 0 stage3 0 stage4 1 stage2dep5 0 stage3dep5 0

stage4dep5 1, atif(dep5==1) atindweights(p04)) ///

lincom(1 1 1 1 -1 -1 -1 -1) lincomvar(tie_ac2) ///

expsurv(using(popmort.dta) ///

datediag(dx) ///

agediag(agediag) ///

pmrate(rate) ///

pmage(age) ///

pmyear(year) ///

pmother(dep sex) ///

at1(dep .) ///

at2(dep .) ///

at3(dep .) ///

at4(dep .) ///

at5(dep .) ///

at6(dep .) ///

at7(dep .) ///

at8(dep .))

By applying expsurv(),the expected mortality rates included in popmort.dta file are incorporated in the contrast and individuals are matched at age at diagnosis (age), calendar year(year), and other characteristics (dep sex). By using options at(dep .) we allow each patient to keep their observed expected survival as opposed to at(dep 1) that would set everyone’s deprivation status to that of the exposed group and would therefore also apply the expected mortality rates of the exposed to everyone.

The avoidable deaths under interventions are derived in a similar way as described above. For example, the avoidable deaths for the most deprived by shifting the stage distribution of the most deprived to that of the least deprived is obtained by including the option per(3228) for the choice of $N^{*}$ equal to 3228 patients :

standsurv, failure timevar(timevar) per(3228) ///

at1(dep5 1 stage2 0 stage3 0 stage4 0 stage2dep5 0 stage3dep5 0

stage4dep5 0, atif(dep5==1) atindweights(p11)) ///

at2(dep5 1 stage2 1 stage3 0 stage4 0 stage2dep5 1 stage3dep5 0

stage4dep5 0, atif(dep5==1) atindweights(p12)) ///

at3(dep5 1 stage2 0 stage3 1 stage4 0 stage2dep5 0 stage3dep5 1

stage4dep5 0, atif(dep5==1) atindweights(p13)) ///

at4(dep5 1 stage2 0 stage3 0 stage4 1 stage2dep5 0 stage3dep5 0

stage4dep5 1, atif(dep5==1) atindweights(p14)) ///

at5(dep5 1 stage2 0 stage3 0 stage4 0 stage2dep5 0 stage3dep5 0

stage4dep5 0, atif(dep5==1) atindweights(p01)) ///

at6(dep5 1 stage2 1 stage3 0 stage4 0 stage2dep5 1 stage3dep5 0

stage4dep5 0, atif(dep5==1) atindweights(p02)) ///

at7(dep5 1 stage2 0 stage3 1 stage4 0 stage2dep5 0 stage3dep5 1

stage4dep5 0, atif(dep5==1) atindweights(p03)) ///

at8(dep5 1 stage2 0 stage3 0 stage4 1 stage2dep5 0 stage3dep5 0

stage4dep5 1, atif(dep5==1) atindweights(p04)) ///

lincom(1 1 1 1 -1 -1 -1 -1) lincomvar(ADb) ///

expsurv(using(popmort.dta) ///

datediag(dx) ///

agediag(agediag) ///

pmrate(rate) ///

pmage(age) ///

pmyear(year) ///

pmother(dep sex) ///

at1(dep 5) ///

at2(dep 5) ///

at3(dep 5) ///

at4(dep 5) ///

at5(dep 5) ///

at6(dep 5) ///

at7(dep 5) ///

at8(dep 5))

In the above prediction, the avoidable deaths are estimated only among the most deprived patients by using atif(dep5==1). As a result, using at1(dep 5) is equivalent to using at1(dep .) within the expsurv() option.
